# Social Media Addiction as a Strong Predictor of Reduced Work Performance

**DOI:** 10.1192/j.eurpsy.2026.10830

**Published:** 2026-07-31

**Authors:** N. Rmadi, R. Affes, N. Kotti, F. Dhouib, M. Hajjaji, K. Jmal Hammami

**Affiliations:** 1 Occupational medicine department, Hedi Chaker university hospital; 2 Family medicine department, Faculty of medicine of sfax, Sfax, Tunisia

## Abstract

**Introduction:**

Social media platforms have become central to communication and networking among healthcare trainees. However, their excessive use may lead to addictive behaviors that compromise professional performance.

**Objectives:**

To assess the association between social media addiction and professional performance.

**Methods:**

A cross-sectional study was carried out in 2025 among 429 residents across four Tunisian medical faculties. Social media addiction was assessed using the Bergen Social Media Addiction Scale (BSMAS), while work performance was measured by the Individual Work Performance Questionnaire (IWPQ). Comparative analyses between addicted and non-addicted residents were conducted.

**Results:**

Social media addiction was observed in 25.6% of residents. Addicted residents demonstrated significantly poorer performance across all IWPQ dimensions. Task performance was lower (2.31 ± 0.80 vs. 2.68 ± 0.77, p<0.001), contextual performance was reduced (2.62 ± 0.82 vs. 2.86 ± 0.80, p=0.007), and counterproductive behaviors were higher (2.72 ± 0.91 vs. 2.40 ± 0.78, p=0.001). Overall performance scores were significantly lower among addicts (2.73 ± 0.49 vs. 3.04 ± 0.48, p<0.001).

**Conclusions:**

Social media addiction exerts a pronounced negative effect on work performance. These results underline the importance of addressing social media overuse in residency programs to protect both professional efficiency and quality of patient care.

**Disclosure of Interest:**

None Declared

